# Solid Pseudopapillary Neoplasm of the Pancreas and Abdominal Desmoid Tumor in a Patient Carrying Two Different *BRCA2* Germline Mutations: New Horizons from Tumor Molecular Profiling

**DOI:** 10.3390/genes12040481

**Published:** 2021-03-26

**Authors:** Andrea Mafficini, Rita T. Lawlor, Claudio Ghimenton, Davide Antonello, Cinzia Cantù, Gaetano Paolino, Alessia Nottegar, Maria L. Piredda, Roberto Salvia, Michele Milella, Angelo P. Dei Tos, Matteo Fassan, Aldo Scarpa, Claudio Luchini

**Affiliations:** 1 Department of Diagnostics and Public Health, Section of Pathology, University and Hospital Trust of Verona, 37134 Verona, Italy; andrea.mafficini@univr.it (A.M.); ritateresa.lawlor@univr.it (R.T.L.); cinzia.cantu@univr.it (C.C.); alessia.nottegar@gmail.com (A.N.); aldo.scarpa@univr.it (A.S.); 2 ARC-Net Research Centre, University and Hospital Trust of Verona, 37134 Verona, Italy; claudio.ghimenton@aovr.veneto.it (C.G.); gaetano.paolino01@gmail.com (G.P.); lilianapiredda@yahoo.it (M.L.P.); 3 Department of Surgery, The Pancreas Institute, University of Verona, 37134 Verona, Italy; davide.antonello@univr.it (D.A.); roberto.salvia@univr.it (R.S.); 4 Department of Medicine, Section of Medical Oncology, University of Verona, 37134 Verona, Italy; michele.milella@univr.it; 5 Department of Medicine (DIMED), Section of Pathological Anatomy, University of Padua, 35121 Padua, Italy; angelo.deitos@unipd.it (A.P.D.T.); matteo.fassan@unipd.it (M.F.)

**Keywords:** *BRCA*, molecular profile, pseudopapillary, pancreatic tumors, desmoid, molecular tumor board

## Abstract

This case report describes the history of a 41 year-old woman with a solid pseudopapillary neoplasm (SPN) of the pancreas and a metachronous abdominal desmoid tumor (DT) that occurred two years after the SPN surgical resection. At next-generation sequencing of 174 cancer-related genes, both neoplasms harbored a *CTNNB1* somatic mutation which was different in each tumor. Moreover, two *BRCA2* pathogenic mutations were found in both tumors, confirmed as germline by the sequencing of normal tissue. The *BRCA2* mutations were c.631G>A, resulting in the amino-acid change p.V211I, and c.7008-2A>T, causing a splice acceptor site loss. However, as the two neoplasms showed neither loss of heterozygosity nor somatic mutation in the second *BRCA2* allele, they cannot be considered as *BRCA*-dependent tumors. Nevertheless, this study highlights the important opportunities opened by extensive tumor molecular profiling. In this particular case, it permitted the detection of *BRCA2*-germline mutations, essential for addressing the necessary *BRCA*-related genetic counseling, surveillance, and screening for the patient and her family.

## 1. Introduction

Solid pseudopapillary neoplasms (SPNs) of the pancreas are rare low-grade malignant neoplasms. In the last WHO classification of digestive system tumors, SPNs were reported among malignant exocrine tumors of the pancreas [1]. Nevertheless, they have been associated with local invasion or distant metastasization only in a small proportion of patients (<5%) [1,2,3]. They almost invariably display somatic activating mutations of the *CTNNB1* gene [4,5,6]. This gene encodes for β-catenin, a crucial protein in biological homeostasis, normally located on the cell membrane. It is involved in transcription and cell-to-cell adhesion [7,8,9]. In different tumor types, including SPNs, Wnt-stimulated β-catenin is translocated into the nucleus, where it acts as a transcriptional co-activator with DNA-binding proteins, ultimately supporting tumor growth and progression [10].

Desmoid tumors (DTs) are considered to be of intermediate malignancy, with potential local aggressiveness and unpredictable clinical course [11,12]. Sporadic DTs are also known for mutations of *CTNNB1* [12]. While SPNs do not belong to any defined genetic syndrome, DTs have already been described in the context of familial adenomatous polyposis (FAP) [12], and these are known to harbor germline mutations of *APC* [13].

Herein, we report the history of a woman with a surgically resected SPN and a metachronous DT that occurred two years after the SPN resection. Targeted next-generation sequencing was performed using formalin-fixed, paraffin-embedded material [14] of both tumors to evaluate the molecular profile and possible association between these seemingly distinct tumors. This study highlights the important role and the new opportunities for personalized medicine provided by tumor molecular profiling. 

## 2. Case Report and Discussion

**Figure 1:**
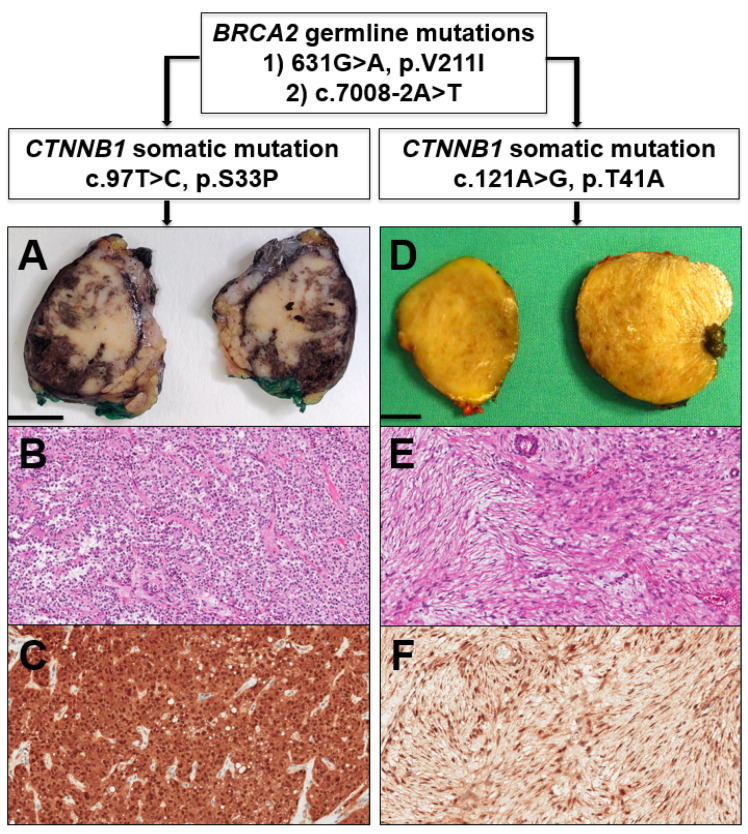
Graphical summary of the presented case. In the genetic background of two different *BRCA2* germline mutations, the patient first developed a solid pseudopapillary neoplasm of the pancreas. (**A**) Gross appearance, scale bar = 1 cm; (**B**) Histology, hematoxylin-eosin staining, original magnification 10×; (**C**) Nuclear positivity for β-catenin at immunohistochemistry, original magnification 10× and, two years after its surgical resection, a desmoid tumor; (**D**) Gross appearance, scale bar = 1 cm; (**E**) histology, hematoxylin-eosin staining, original magnification 10×; (**F**) Nuclear positivity for β-catenin at immunohistochemistry, original magnification 10×, each with a different *CTNNB1* somatic mutation.

A graphical summary of this case report is provided in Figure 1.

A 41 year-old female with an unremarkable medical history underwent a medical examination fora vague abdominal discomfort and asthenia for three months. She had no cancer-related familial history, except for the paternal grandmother who died of esophageal carcinoma. No other cancer-related events were recorded in the family history. A computed-tomography (CT) scan revealed the presence of a well-circumscribed mass of about 3 cm in the pancreatic body, without evidence of distant metastasization. Based on this finding and the histopathological diagnosis based on material from endoscopic ultrasound-guided fine-needle aspiration (EUS-FNA), which supported a diagnosis of SPN vs. neuroendocrine tumor, the patient underwent robot-assisted distal pancreatectomy with surgical resection of the pancreatic mass. At the first intraoperative frozen section analysis, the pancreatic margin was involved with few neoplastic cells, thus the resection was extended (1 cm). The successive pancreatic margin, also examined on the frozen section, was considered negative. Globally considered, the pancreatic mass was surgically resected with free margins. The subsequent gross sampling revealed a brownish tumor with pushing borders, mainly located in the central-posterior portion of the pancreatic body. Histologically, the tumor showed a mixture of solid, pseudopapillary, and cystic-hemorrhagic areas. Solid areas included poorly cohesive, monomorphic, and polygonal cells, intermingled with a rich net of blood vessels. At the same time, in the pseudopapillary areas, neoplastic elements showed focal detachment and the remaining cells were left to surround the blood vessels, resembling papillary structures. Neoplastic cells presented an eosinophilic cytoplasm and monomorphic roundish nuclei with granular chromatin, without an evident nucleolus. Mitotic figures were absent. Although at grossing the tumor mass seemed very well-demarcated, at microscopy, the periphery tumor cells showed a focal invasion of adjacent pancreatic parenchyma and adipose tissue. Immunohistochemistry (IHC) showed that neoplastic elements were positive for β-catenin (nuclear positivity), CD10, progesterone receptor, vimentin, and LEF1, and were negative for Bcl-10, trypsin, and chromogranin-A. The histological examination, coupled with immunohistochemical analysis, was conclusive for a diagnosis of solid pseudopapillary neoplasm (SPN) of the pancreas.

**Figure 2:**
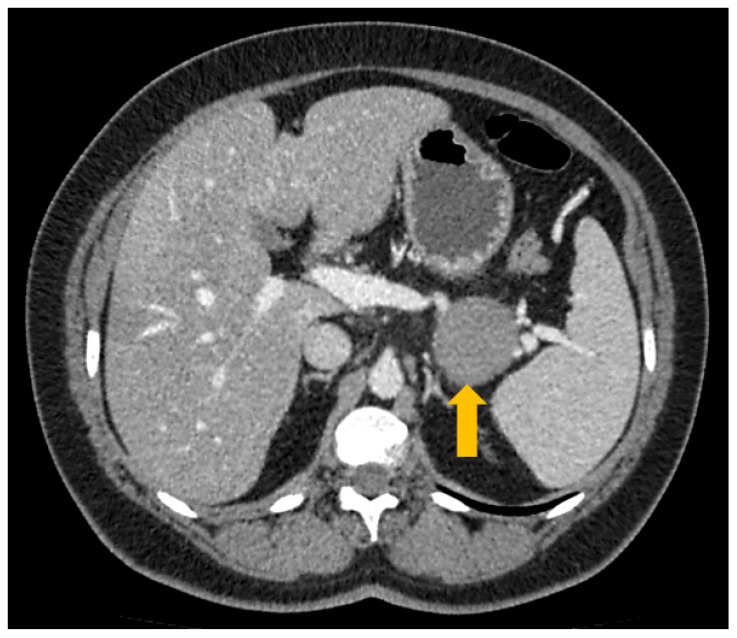
CT scan showing the presence of a roundish mass (4.5 cm, yellow arrow) between the stomach and the spleen.

One year after the surgical resection, the patient developed type II diabetes, which was treated with oral hypoglycemic therapy. The standard follow-up, comprised of blood tests and a physical examination, was performed every 12 months but did not reveal any alteration. During an imaging-based follow-up two years after the pancreatic resection, magnetic resonance imaging (MRI) revealed the presence of a roundish mass of about 4 cm located in the epiploon retrocavity, strictly adjacent to the site of previous surgical resection. A following CT-scan confirmed the presence of the mass, which showed a homogeneous density and a maximum diameter of about 4.5 cm (Figure 2). 

With the suspicion of a relapse of the SPN vs. an inflammatory pseudotumor due to the previous surgery and based on the histopathological diagnosis predicated on material from EUS-FNA, which cannot rule out a relapse of SPN (cells positive for β-catenin), the patient underwent a second operation with the surgical resection of the abdominal mass. The subsequent gross sampling revealed the presence of a roundish nodule; on the cut surface, it was yellowish and showed homogeneous features without necrotic or hemorrhagic areas. Histologically, the mass appeared as a well-circumscribed tumor, arranged in intersecting fascicles. Neoplastic cells were spindle-shaped with elongated nuclei and bipolar cytoplasm with blunt ends. Mitotic activity was virtually absent (less than 1 out of 50 high-power fields). IHC showed tumor cells to be diffusely positive for β-catenin (nuclear positivity), CD10, and vimentin, focally positive for CD99 and LEF1, and negative for S100, STAT6, MDM2, Bcl-2, CD34, CD117, DOG1, smooth-muscle actin, desmin, and progesterone receptor. The histopathological and immunohistochemical features were consistent with the diagnosis of desmoid tumor.

Given the peculiar association of SPN and DT in the same patient, these neoplasms, as well as a normal tissue sample (spleen), were further examined by targeted next-generation sequencing of 174 cancer-associated genes. 

Both the SPN and DT were microsatellite stable and showed a low tumor-mutation burden (SPN 5.6 mutations/Mb; DT 6.1 mutations/Mb). Somatic mutations were only found in the *CTNNB1* gene, while a double germline mutation was detected in the *BRCA2* gene.

Both SPN and DT harbored a somatic mutation of the *CTNNB1* gene, but each neoplasm carried a different mutation. SPN featured a heterozygous c.97T>C transition, which causes a missense p.S33P mutation resulting in enhanced activity of the *CTNNB1* gene product [15]. This variant falls within a known mutational hotspot of the *CTNNB1* gene that has been reported in multiple solid tumors [16]. It is registered in dbSNP (rs105719886) and is pathogenic according to ACMG/AMP criteria [17]. DT showed a somatic heterozygous c.121A>G transition, which leads to a missense p.T41A resulting in enhanced activity of the *CTNNB1* gene product [18,19,20,21]. This variant is also located in a mutational hotspot of the gene and has been reported in multiple solid tumors [22,23]. It is registered in dbSNP (rs121913412) and is pathogenic according to the ACMG/AMP criteria [17]. 

Sequence analysis of tumors and normal tissue showed the presence of two heterozygous germline variants in the *BRCA2* gene. Both variants are classified as pathogenic according to the ACMG/AMP criteria [17] and the ClinVar database (https://www.ncbi.nlm.nih.gov/clinvar/, accessed on 18 October 2020). Notably, there was neither loss of heterozygosity (LOH) nor any somatic mutation in the second *BRCA2* allele of both tumor samples. The first variant of the *BRCA2* gene was the c.631G>A transition of the last nucleotide of exon 7. This causes both the p.V211I missense variant and the disruption of a natural splice donor site, which results in abnormal splicing [24]. This variant is classified as pathogenic in the ClinVar database (accession VCV000052058). The second *BRCA2* variant was the c.7008-2A>T transversion of the penultimate nucleotide of intron 13 that alters a natural splice acceptor site, resulting in abnormal splicing and non-functional transcripts [25]. This variant is classified as pathogenic in the ClinVar database (accession VCV000052246). 

After discussion in the multidisciplinary molecular tumor board [26], given the presence of *BRCA2* germline mutations, the patient was enrolled in screening surveillance and genetic counseling following existing guidelines [27,28]. A discussion also focused on a possible interconnection between *BRCA2* and *CTNNB1* mutations. However, on the one hand, *BRCA* genes (tumor suppressors)/the encoded proteins belong to the homologous recombination (HR) DNA repair machinery [14], and on the other hand, the *CTNNB1* gene (oncogene)/the encoded protein β-catenin, belong to the Wnt pathway [7,8,9]. Currently, there is no evidence of any interconnection of these two pathways/mechanisms, and our report (*CTNNB1* activating mutation, mono-allelic *BRCA2* mutation) is in line with this knowledge.

## 3. Materials and Methods

This study was conducted in accordance with the Good Practice guidelines, the Declaration of Helsinki, and current laws, ethics, and regulations, after registering the informed consent signed by the patient.

### 3.1. Immunohistochemistry

SPN and desmoid tumors were extensively analyzed with immunohistochemical analysis, using standardized procedures as already described [29,30]. Briefly, 4 μm formalin-fixed paraffin-embedded sections were immunostained with selected antibodies. Heat-induced antigen retrieval was performed using a heated plate and 0.01 mol/L of citrate buffer, pH 8.9, for 15 min. Light nuclear counterstaining was performed with hematoxylin. All samples were processed using a sensitive peroxidase-based “Bond Polymer Refine” detection system in an automated Bond instrument (Vision-Biosystem, Leica, Milan, Italy). Sections incubated without the primary antibody served as negative controls. The following antibodies were used for SPN diagnosis (and for the differential diagnosis with neuroendocrine tumors and acinar cell carcinoma): β-catenin (clone: 15B8, dilution: 1:400, Source: Sigma Aldrich, St. Louis, MO, USA), CD10 (56C6, 1:50, Novocastra, Newcastle, UK), progesterone receptor (PgR 636, 1:150, Dako, Santa Clara, CA, USA), vimentin (V9, 1:50, Novocastra), LEF1 (EPR2029Y, 1:200, Novus-Abcam, Cambridge, UK), Bcl-10 (331.3, 1:1000, Santa Cruz, Dallas, TX, USA), trypsin (polyclonal rabbit, 1:500, Tema Ricerca, Bologna, Italy), and chromogranin-A (DAK-A3, 1:2500, Dako). In addition to these, other antibodies were used for supporting the diagnosis of DT (vs. other spindle-cell proliferations/neoplasms) as follows: CD99 (O13, 1:400, DBA Covance, Princeton, NJ, USA), S100 (polyclonal rabbit, 1:3000, Dako), STAT6 (two different clones: phospho Y64I, 1:100, Novus-Abcam, and D1, 1:200, Santa Cruz), MDM2 (IF2, 1:400, Invitrogen, Carlsbad, CA, USA), Bcl-2 (Ab692, 1:100, Novus-Abcam), CD34 (QBEND/10, 1:200, Novocastra), CD117 (polyclonal rabbit, 1:100, Dako,), DOG1 (K9, 1:100, Novocastra), smooth-muscle actin (1A4, 1:250, Dako), and desmin (D33, 1:1000, Dako).

### 3.2. Molecular Analysis

SPN and desmoid tumors, as well as a normal tissue specimen (spleen), were analyzed with molecular analysis using next-generation sequencing.

#### 3.2.1. DNA Extraction

Genomic DNA was extracted from formalin-fixed paraffin-embedded (FFPE) tissues using the GeneRead DNA FFPE kit (Qiagen, Hilden, Germany) according to the manufacturer’s instructions. The kit procedure included the removal of deaminated cytosine to prevent false results in DNA sequencing. Neoplastic cellularity was evaluated by two pathologists (C.L., B.R.) on hematoxylin and eosin staining, and each tumor sample was manually microdissected with a fine-needle hypodermic syringe to enrich for tumor cells. Quantification of genomic DNA samples was performed with the Qubit dsDNA HS assay kit on a Qubit fluorometer (ThermoFisher, Waltham, MA, USA) and qualification was done as previously described [31].

#### 3.2.2. Massive Parallel Sequencing (Next-Generation Sequencing, NGS)

NGS was performed using the SureSelectXT HS CD Glasgow Cancer Core assay (www.agilent.com, accessed on 20 October 2020), hereinafter referred to as CORE. The panel spans 1.8 megabases of the genome and interrogates 174 genes for somatic mutations, copy number alterations, and structural rearrangements; the detail of targeted genes is reported in Supplementary Table S1. Sequencing libraries were prepared by targeted capture using the SureSelect kit (Agilent Technologies, Santa Clara, CA, USA), with RNA baits targeting a bespoke set of selected genomic features. Briefly, 10–100 ng of genomic DNA extracted from FFPE tissue was enzymatically fragmented with the SureSelect Enzymatic Fragmentation Kit (Agilent Technologies, Santa Clara, CA, USA). Overhanging DNA fragments were subsequently end-repaired, adenylated, ligated to indexing/sequencing adapters, enriched by PCR, and purified following the manufacturer’s instructions. The quality and quantity of purified pre-capture libraries were assessed using the Qubit BR dsDNA assay (ThermoFisher, Waltham, MA, USA). Hybridization-capture and purification of the libraries were performed according to the manufacturer’s instructions. Using 1.6 µg of pooled DNA from 16 pre-capture libraries, 100 ng from each pre-capture library was used to prepare 16-library pools. Captured library pools were enriched by PCR, purified, and quantified using the Qubit dsDNA HS assay. The quality of the library pools was verified with the Agilent 4200 Tape Station and High Sensitivity D1000 ScreenTape (Agilent Technologies, Santa Clara, CA, USA). Sequencing was performed on a NextSeq 500 (Illumina, San Diego, CA, USA) loaded with 2 captured library pools, using a high-output flow cell and 2 × 75 bp paired-end sequencing.

CORE panel analysis started with demultiplexing performed with FASTQ Generation v1.0.0 on the BaseSpace Sequence Hub (https://basespace.illumina.com, accessed on 23 August 2020). Forward and reverse reads from each demultiplexed sample were aligned to the human reference genome (version hg38/GRCh38) using BWA and saved in the BAM file format [32]. BAM files were sorted, subjected to PCR duplicate removal, and indexed using biobambam2 v2.0.146 (https://gitlab.com/german.tischler/biobambam2.git; accessed on 30 November 2020) [33]. Coverage statistics were produced using SAMtools [34]. Calling of all variant types (small nucleotide variants, copy number variations, and structural variants) was performed on the tumor samples using a set of 20 non-neoplastic samples as a reference. These samples were retrieved at our institution, processed, and sequenced with the same workflow to yield comparable BAM files. 

Single-nucleotide variants were called using Shearwater [35]. Small (<200 bp) insertions and deletions were called using Pindel [36]. Small nucleotide variants were further annotated using a custom pipeline based on vcflib (https://github.com/ekg/vcflib; accessed on 30 November 2020), SnpSift [37], the Variant Effect Predictor (VEP) software [38], and the NCBI RefSeq transcripts database (https://www.ncbi.nlm.nih.gov/refseq/; accessed on 30 November 2020). Annotated variants were filtered keeping only missense, nonsense, frameshift, or splice site variants as annotated from the canonical transcripts. In case of alternative exon usage between transcripts, the transcript where the variant produced the worst effect was retained and used for annotation. All candidate mutations were manually reviewed using Integrative Genomics Viewer (IGV), version 2.4 [39], to exclude sequencing artefacts. 

Tumor mutational burden for each sample was computed as follows: insertions, deletions, complex variants, and variants with an allelic frequency below 5% were removed from the variant list. Variants with more than 10 entries in the GnomAD database (https://gnomad.broadinstitute.org/; accessed on 30 November 2020) were removed as potential germline variants. The remaining variants, including synonymous, non-synonymous, intronic, and splice site variants, were counted and normalized for the footprint of the panel (1.85 megabases) to obtain the number of mutations per megabase. 

Microsatellite instability was computed following the method of Papke et al. [40]. Briefly, called variants were filtered to retain only insertions and deletions detected in adenine and thymine mononucleotide repeats of length between 4 and 12 base pairs. Variants were further filtered to remove those with more than 10 entries in the GnomAD database as potential germline variants, counted, and normalized to obtain the number of mutations per megabase.

Copy number alterations of targeted genes were detected using the geneCN software, developed at Wolfson Wohl Cancer Research Centre (https://github.com/wwcrc/geneCN; accessed on 31 October 2020), and the set of 20 non-neoplastic samples retrieved at our institution as a reference baseline for diploid DNA. Whole-chromosome or chromosome-arm alterations were assessed by measuring the ratio of normalized, GC-adjusted coverage of tumor samples’ alignments to the mean, normalized, and GC-adjusted coverage of 20 non-neoplastic samples for all targeted regions of a chromosome arm. Targeted regions included both targeted genes and a set of “backbone” regions, probing each chromosome at 1 megabase intervals. Each large alteration was further confirmed by checking the copy number status of targeted genes included in the large alteration itself, as reported by the geneCN software.

Structural rearrangements were detected using the BRASS software [41], and visually reviewed using Integrative Genomics Viewer (IGV), version 2.4 [39] to exclude sequencing artefacts.

#### 3.2.3. Variant Classification

Variants were classified following the five-tier classification system recommended by the joint consensus of the American College of Medical Genetics and Genomics and the Association for Molecular Pathology (ACMG/AMP) [17]. Variants were thus classified as Benign (class 1), Likely Benign (class 2), Variant of Uncertain Significance (VUS—class 3), Likely Pathogenic (class 4), and Pathogenic (class 5). The variant classification was retrieved from the ClinVar database when available (https://www.ncbi.nlm.nih.gov/clinvar/; accessed on 16 November 2020) and accepted when the record complied with the following requisites: reviewed by an expert panel according to the ACMG/AMP guidelines and/or reported by multiple submitters with evaluation criteria according to the ACMG/AMP guidelines with no conflicts. When a consistent classification was unavailable or when the variant was not present in the ClinVar database, variants were evaluated in-house, according to the ACMG/AMP guidelines while using the following databases and software to gather and integrate all relevant information: My Cancer Genome (https://www.mycancergenome.org; accessed on 20 April 2020), Intogen [42] (https://www.intogen.org/search; accessed on 23 November 2020), and QIAGEN Clinical Insight (QCI) software (https://variants.qiagenbioinformatics.eu/qci/; accessed on 16 November 2020).

## 4. Conclusions

We report the case of a middle-aged woman carrying two *BRCA2* germline mutations, who developed a pancreatic SPN, followed by a DT arising 2 years after the pancreatic resection. Both the SPN and DT harbored different *CTNNB1* somatic mutations. Although the SPN and DT showed neither LOH nor any somatic mutation in the second *BRCA2* allele and thus cannot be considered as *BRCA*-dependent tumors, this study highlights the important role and the new opportunities derived from tumor molecular profiling. Indeed, it allowed the detection of two *BRCA2*-germline mutations, which was essential for addressing the necessary screening, surveillance, and genetic counseling for the patient.

## Data Availability

All data are available in the paper.

## Supplementary Materials

The following are available online at https://www.mdpi.com/article/10.3390/genes12040481/s1, Supplementary Table S1. Details of targeted genes of CORE panel.
