# Supplementary material for: Solid Pseudopapillary Neoplasm of the Pancreas and Abdominal Desmoid Tumor in a Patient Carrying Two Different BRCA2 Germline Mutations: New Horizons from Tumor Molecular Profiling
Source: Genes (Basel). 2021 Mar 26;12(4):481. doi: 10.3390/genes12040481 (PMC8065547; doi:10.3390/genes12040481)
Supplement: Supplementary file 1 [file genes-12-00481-s001.pdf]

Supplementary Table 1: Targeted genes in the CORE sequencing assay

Genes covered for all coding exons

AKT1	AKT2	AKT3	ALK	AMER1	APC	APLNR	AR	ARAF	ARID1A	ARID1B
ARID2	ASXL1	ATM	ATR	ATRX	B2M	BAP1	BLM	BRAF	BRCA1	BRCA2
CBL	CCND1	CCND3	CD274	CD58	CDK12	CDK4	CDKN1A	CDKN1B	CDKN2A	CDKN2B
CHEK2	CIITA	CREBBP	CTCF	CTNNB1	DAXX	DICER1	DNMT3A	EGFR	EP300	EPHA3
ERBB2	ERBB3	ERBB4	ERG	ESR1	ETV6	EZH2	FAS	FBXW7	FGFR1	FGFR2
FGFR3	FGFR4	GATA3	GNA11	GNAQ	GNAS	H3F3A	H3F3B	HIST1H3B	HIST1H3C	HIST2H3C
HLA-A	HLA-B	HLA-C	HNF1A	HRAS	IDH1	IDH2	JAK1	JAK2	JAK3	JUN
KDR	KIT	KLF4	KMT2A	KRAS	MAP2K1	MAP2K2	MAP2K4	MAP3K1	MAPK1	MAX
MED12	MEN1	MET	MLH1	MSH2	MSH6	MTOR	MUTYH	MYB	MYC	MYCN
NBN	NF1	NF2	NFE2L2	NOTCH1	NOTCH2	NOTCH3	NOTCH4	NPM1	NRAS	NTRK1
PALB2	PBRM1	PDCD1LG2	PDGFRA	PDGFRB	PHF6	PIK3CA	PIK3CB	PIK3R1	PMS2	POLE
POLQ	PPP2R1A	PTCH1	PTEN	PTPN11	RAC1	RAD21	RAD50	RAF1	RB1	RET
RHOA	RNF43	ROS1	RPL5	RUNX1	SETBP1	SETD2	SF3B1	SMAD4	SMARCA4	SMARCB1
SMO	SOCS1	SPOP	STAG1	STAG2	STAT3	STAT5B	STK11	SYK	TGFBR2	TP53
TSC1	TSC2	U2AF1	VHL	WT1						

Cancer CORE: genes covered for copy number alterations

AKT1	AKT2	AKT3	ALK	APC	APLNR	AR	AURKA	AXL	B2M	BCL2
BRAF	BRCA1	BRCA2	CCND1	CCND2	CCND3	CCNE1	CD274	CDK2	CDK4	CDK6
CDKN1B	CDKN2A	CDKN2B	CIITA	CTNNB1	EGFR	EPHA3	ERBB2	ERG	ESR1	ETV6
EZH2	FGF19	FGFR1	FGFR2	FGFR3	FGFR4	HGF	HLA-A	HLA-B	HLA-C	IGF1R
JAK1	JAK2	JAK3	JUN	KIT	KRAS	MCL1	MDM2	MET	MLH1	MYC
NFE2L2	NOTCH1	NOTCH2	NOTCH3	NOTCH4	NTRK1	PALB2	PDCD1LG2	PDGFRA	PDGFRB	PIK3CB
PTEN	RAF1	RB1	RET	SMAD4	SOCS1	SRC	STAT3	SYK	TERT	TP53
TSC1	TSC2	VHL	YAP1							

Cancer CORE: genes with full-gene footprint for structural variations

APLNR	B2M	BRCA1	BRCA2	CDKN2A	CDKN2B	HLA-A	HLA-B	HLA-C	PTEN	RB1
TP53	TSC1									

Cancer CORE: genes covered for fusions detection

ALK	BRAF	EGFR	ETV6*	FGFR2	FGFR3	NTRK1	RAF1	RET	ROS1	TERT

*ETV6 reports NTRK3 fusions
